# Increasing Awareness and Reducing Occupational Hazards in the Cardiac Catheterization and Electrophysiology Laboratories: Working to Eliminate Lead and Complications of Radiation in Cardiovascular Team Study

**DOI:** 10.1016/j.jacadv.2025.102496

**Published:** 2026-01-23

**Authors:** Estelle Torbey, Suleman Ilyas, Kamala Tamirisa, Yongkang Zhang, Christopher Steelman, Phinnara Has, Karen Aspry, Jim W. Cheung, ACC EP Leadership Council, ACC Interventional Leadership Council; ACC Women in Cardiology Council; ACC Cardiovascular Team Council; Rhode Island, Texas, and New York ACC Chapters

**Affiliations:** a Division of Cardiology, Department of Medicine, Brown University, Providence, Rhode Island, USA; b Division of Cardiology, Department of Medicine, Texas Cardiac Arrhythmia Institute, Austin and Dallas, Texas, USA; c Department of Medicine, Brown University, Providence, Rhode Island, USA; d Cardiac Specialist Program, Weber State University, Ogden, Utah, USA; e Department of Biostatistics, Epidemiology, Research Design and Informatics Core, Brown University Health, Providence, Rhode Island, USA; f Division of Cardiology, Department of Medicine, Weill Cornell Medicine, New York, New York, USA

**Keywords:** cardiac catheterization lab, fluoroless, occupational hazards

## Abstract

**Background:**

Increasing procedural volumes in the electrophysiology and cardiac catheterization laboratories (EP/CCLs) expose physicians, staff, and nurses to hazards from ionizing radiation and prolonged lead-apron use.

**Objectives:**

To characterize the adverse health outcomes among contemporary EP/CCL workers and to evaluate an educational intervention to mitigate risk.

**Methods:**

Two surveys were distributed 3 months apart to the American College of Cardiology chapters and assessed injuries and ailments, perceived risk awareness, and knowledge of potential interventions to mitigate harm. The initial survey was paired with a video on ergonomic strategies to prevent musculoskeletal (MSK) injury, radiation safety principles, and best practices for pregnant workers. Univariable logistic regression models were used to assess associations between each baseline variable separately and health outcomes related to occupational hazards.

**Results:**

Of the 306 initial respondents, 70% were 31 to 60 years old, and 43% were women. Thirty-six percent were interventional cardiologists, 30% were nonphysician cardiovascular team members, 64% had >10 years' EP/CCL experience, and 43% wore lead >8 hours/day. Risk factors for MSK pain/injury included age ≥50 (adjusted OR [aOR]: 2.19; 95% CI: 1.15 to 4.16; *P* = 0.02), lead-apron use >4 hours/day (aOR: 2.39; 95% CI: 1.31-4.39; *P* = 0.05), and limited knowledge of mitigation strategies (aOR: 4.62; 95% CI: 1.62-11.45; *P* = 0.003). Among women with prior pregnancy, 41.5% reported worsening MSK discomfort due to occupational duties; 52.1% lacked opportunities to adjust procedural load; and 76% could not reduce work hours. On follow-up, 75.9% reported adopting ergonomic techniques from the video.

**Conclusions:**

Occupational hazards remain prevalent in the modern EP/CCL. Focused educational interventions may help to reduce injuries and risks.

Procedural volumes for electrophysiology (EP) and interventional cardiology (IC) are increasing as new technologies help treat patients with cardiovascular disease of increasing complexity.^1^ This leads to increased exposure to ionizing radiation and prolonged lead wearing, which can result in injuries, including musculoskeletal (MSK) ailments, radiation-induced damage to the lens of the eye, and possible association with increased malignancy risk and pregnancy complications.^2^, ^3^, ^4^ Prior studies have reported the deleterious effects of both ionizing radiation and lead aprons in the EP/cardiac catheterization laboratory (CCL).^5^, ^6^, ^7^, ^8^ Guidance on prevention and treatment remains limited, as reported in these studies. The effects of chronic ionizing radiation can be mitigated with proper education and standardized policies, which can reduce exposure by up to 90%.^5^^,^^6^ This issue is further compounded by the growing number of female operators working in EP/CCL during gestation.^3^^,^^9^ Our study sought to evaluate the current prevalence of various medical conditions that may arise among EP/CCL professionals, to identify risk factors (Central Illustration), and to assess the impact of an educational video focused on mitigating these risks. By assessing injury rates, medical complications, and the impact of the educational intervention, the study seeks to inform strategies that improve workplace safety and support the well-being of health care professionals in the EP/CCL.

**Figure 1:**
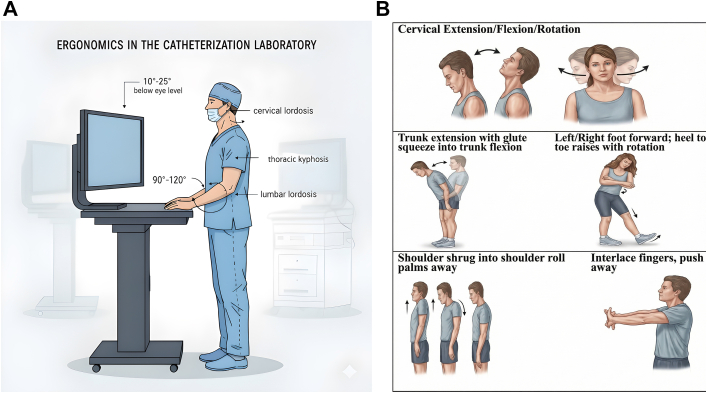
Proposed Ergonomic Adjustments and Exercises in the Cardiac Catheterization Laboratories (A) Ergonomic adjustments in the electrophysiology/Cath cardiac catheterization laboratory. These include supporting a neutral spine, ensuring the lordotic curve of the cervical and lumbar spine, as well as the kyphotic curve of the thoracic spine are maintained. The height of monitors should ideally be below eye level, at 10 to 25°, and directly in front of the operator. Table height should ideally be at the pubic level, which allows for relaxation of arms at the side, with elbows at 90 to 120°. (B) Illustration of stretching exercises. Each exercise is best performed 3 times and include: cervical extension, flexion, and rotation; shoulder shrug into shoulder roll; trunk extension and flexion with and without toe raise. The exercises were assembled to ensure that the total stretching time does not exceed 30 seconds to avoid prolonged work interruption.

**Central Illustration:**
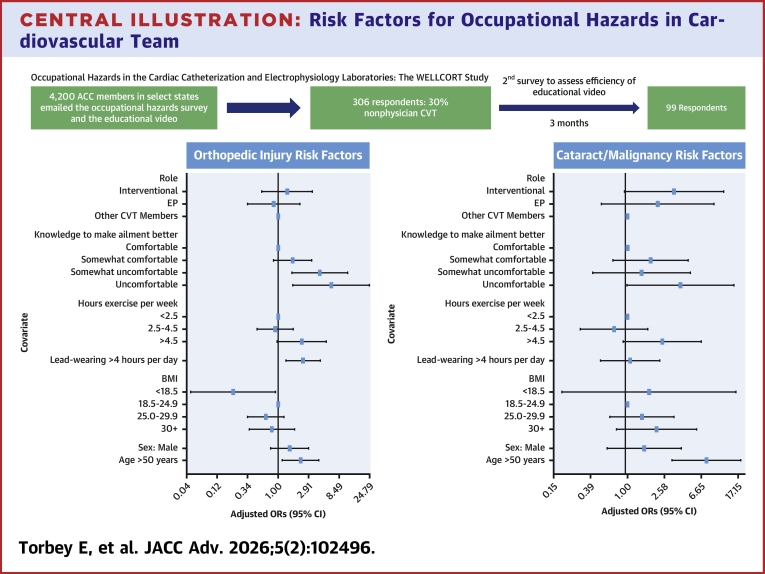
**Risk Factors for Occupational Hazards in Cardiovascular Team** The study comprised of a survey sent out to American College of Cardiology members in collaborating states with an educational video to prevent and manage orthopedic injuries as well as for radiation protection strategies. The 2 Forest plots represent the adjusted ORs for relevant characteristics stemming from the questions surveys that contribute to either orthopedic injuries or cataract/cancer development in electrophysiology/cardiac catheterization laboratory staff exposed to radiation and lead wearing. ACC = American College of Cardiology; BMI = body mass index; CVT = cardiovascular team; EP = electrophysiology.

## Methods

The CV-WELLCORT (Working to Eliminate Lead and Complications of Radiation in cardiovascular Team) project was funded by a seed grant from the ACC. An initial survey ending with an educational video was distributed to the American College of Cardiology (ACC) members from participating states and councils. Three months later, a follow-up survey was sent out to assess the impact of the video. The initial survey was conducted to ascertain the injuries and medical conditions occurring in EP/CCL. At the end of the survey, participants viewed an educational video discussing radiation safety, best practices to prevent orthopedic injuries, implementation of ergonomic interventions, and practical considerations during pregnancy. Surveys were sent to physicians, nurses, and allied health professionals in the participating ACC chapters’ mailing list. The initial survey remained open for 2 months. Data collected included age, subspecialty, sex, body mass index (BMI), years of practice, gestational status, types of procedures performed, mean estimated time of exposure to fluoroscopy, previous injuries or medical complications related to radiation exposure or lead wearing, knowledge about optimal ergonomic measures during procedures, and willingness to implement ergonomic changes. Weekly reminders were sent out to complete the survey anonymously. Three months after reviewing the video, participants were emailed a follow-up survey to assess changes in knowledge and practices regarding orthopedic injury prevention and fluoroscopy exposure reduction, perceived usefulness of the educational video, and intentions to incorporate the learned techniques into practice. Participants also reported any changes in the prevalence of occupational injuries or medical complications. The primary endpoint was the occurrence of adverse events resulting from work-related injuries as reported in the survey. An adverse event was defined as any self-reported cancer, orthopedic injury (with or without subsequent surgery), head injury, or needlestick injury that occurred during work or was believed by the respondent to be attributable to work activities. In addition, pregnancy-related complications that respondents identified as related to or exacerbated by work were included as adverse events. These included MSK injury, syncope, miscarriage, and uterine bleeding.

Participation was voluntary, and all data collected were anonymized and securely stored. The anonymous survey was funded by the ACC grant to the Rhode Island ACC chapter through submission from members of the EP leadership council and with collaboration of the Texas ACC Chapter and New York Chapter ACC and various leadership councils within ACC (women in cardiology, cardiovascular team (CVT), and IC). This manuscript did not require submission to the Institutional Review Board because the project involved an anonymous staff survey conducted solely for quality improvement purposes derived from the ACC grant seed projects to local chapters. No patient data were collected or analyzed, and no individually identifiable information was obtained from participants. The activity posed no more than minimal risk, as defined by federal regulations, and was intended to improve medical staff wellness. Therefore, it met the criteria for a quality improvement initiative that does not constitute human subjects research requiring Institutional Review Boar review.

### Educational video intervention

A 20-minute video accompanied the initial survey consisting of 3 different components. The first part discussed the risks of lead apron use and strategies for optimal ergonomics in the EP/CCL to reduce harm. This was created in collaboration with a physical therapist who emphasized best practices to minimize lead apron-related injuries, including practical ergonomic interventions (Figure 1A). Stretching before and after procedures can assist with pain, and microbreaks are encouraged at 30 to 40 minutes intervals^.^ Thirty-second stretches can be performed during these microbreaks (Figure 1B), including cervical rotation, cervical flexion and extension, shoulder shrugging, midback stretches, lower-back extensions, and ankle flexion and extension that can be performed during work hours in EP/CCL without compromising procedural sterility. The second part discussed general radiation-protection knowledge and strategies to reduce radiation exposure, including decreasing the frame rate, avoiding steep angulation, using disposable radiation shields, wearing lead-protective glasses, using under-table shields, as well as newer shielding technologies. The final part of the video focused on radiation safety, offering data-driven and practical tips during pregnancy.

### Statistical analysis

Univariable logistic regression models were used to assess associations between each baseline variable separately and outcomes of occupational exposure to radiation, lead-apron use, and potential health risks. Variables that were either statistically significant or were deemed of significant clinical importance were included in multivariable logistic regression models to adjust for potential confounding factors. Collinearity between the variables that were included in the multivariable models was assessed by calculating variance inflation factors for each variable, with values ≥ 10 indicating collinearity. The variance inflation factor values from our models ranged from 1.04 to 1.22, indicating the covariates were not collinear. Fisher exact test was used to conduct comparisons between physicians and nonphysician CVT groups as well as between general cardiology respondents, who were considered controls given little time in the EP/CCL, and other respondents. Hosmer-Lemeshow goodness-of-fit test was performed to check for overfitting for each regression model with *P* > 0.05 indicating a good fit. All models exhibited acceptable fitness and area under the curves All tests were two-sided, and a *P* value of <0.05 was considered statistically significant. To control for type I error due to multiple testing, Bonferroni corrections were applied to the significance levels. All analyses were performed using Stata/MP 18.0.

## Results

### Baseline demographics

There were 4,200 ACC members in the Texas, Rhode Island, and New York chapters to whom the surveys were sent. Table 1 details the baseline characteristics of the study population. Of 306 initial respondents, 70% were 31 to 60 years old and 43% were women. Thirty-six percent were interventional cardiologists, 30% were nonphysician CVT members, 64% had >10 years’ EP/CCL experience, and 43% wore lead >8 hours/day. 55% had a BMI >25 kg/m^2^ and 30% reported performing fluoroless procedures.

**Table 1:** Baseline Demographics

Age, y	
20-30	6.7
31-40	23.3
41-50	24.3
51-60	23.3
61-70	13.7
71-80	6.7
Sex	
Male	57
Female	43
BMI, kg/m^2^	
*<*18.5	2.6
18.5-24.9	42.6
25.0-29.9	37.4
>30.0	19.2
Profession	
GC	6.9
Imaging	4.6
EP	18.6
IC	36.6
S-IC	1.3
HF	1.6
AHP	11.1
RN	19.2
Experience, y	
0-5	17.3
5-10	18.6
>10	64
Daily hours of lead wearing	
*<*2	7.2
2-4	15.4
4-8	26.6
>8	43.8
N/A	6.9
Hours of exercise per week	
*<*2.5	40
2.5-4.5	38.9
>4.5	20.1
Percentage of fluoroless procedures	
*<*10	68.2
>20	8.7
*>*50	7.3
N/A	14.7

Values are %. This table represents the baseline characteristics of the survey cohort consisting of 306 responses with 55% respondents wearing lead >4 hours per day; 60% of those with orthopedic injury wore lead for >4 hours compared to 45% of those without orthopedic injury (*P* < 0.02 by Fisher test).

AHP = allied health professional; BMI = body mass index; EP = electrophysiology; GC = general cardiology; HF = heart failure; IC = interventional cardiology; S-IC = structural interventional cardiology; N/A = not applicable; RN = registered nurse.

### Musculoskeletal injury

Neck, knee, back, ankle, or shoulder pain occurred in 62% of respondents among which 7.5% required surgery for their orthopedic ailments (Table 2). Among the risk factors considered, age>50, the use of a lead apron for >4 hours/day and not having the knowledge to alleviate ailments were found to be associated with increased likelihood of MSK pain and injuries (OR: 1.61 [1.01-2.58], *P* = 0.047; OR 1.83 [1.12-2.98], *P* = 0.02; OR 3.32 [1.42-7.74], *P* = 0.003, respectively) (Table 3). In the multivariable model assessing risk factors for orthopedic injury, age >50 and lead wearing >4 hours/day were independently associated with orthopedic injury (adjusted OR [aOR] = 2.19; 95% CI: 1.15-4.16; *P* = 0.02, aOR = 2.39; 95% CI: 1.31-4.39; *P* = 0.005) (Table 4).

**Table 2:** Reported Occupational Injuries and Health Outcomes

Ailments and Injuries	Yes (%)
Cataracts or any skin cancer or orthopedic cancer	16.7
Cancer of the brain, breast, or lung	3.5
Work-related head injury	28.0
Prior needlestick injury	68.8
History of neck, knee, back, ankle, or shoulder pain	62.0
Surgery for an orthopedic injury	7.5
Resolution of orthopedic injuries	50.3

This table represents a summary of the percentages of occupational and procedure-related injuries specific to the electrophysiology/cardiac catheterization laboratory.

**Table 3:** Univariable Regression Models for Orthopedic Injuries

Age, y	
20-30	Reference
31-40	1.58 (0.59, 4.19)
41-50	1.43 (0.54, 3.78)
51-60	2.72 (1.01, 7.38)
61-70	1.86 (0.64, 5.35)
71-80	1.79 (0.52, 6.12)
Age, y	
≤50	Reference
>50	1.61 (1.01, 2.58)
Male	1.31 (0.82, 2.08)
BMI, kg/m^2^	
<18.5	Reference
18.5-24.9	5.73 (1.11, 29.64)
25.0-29.9	4.88 (0.94, 25.36)
≥30	3.69 (0.68, 19.9)
Role	
General cardiology	Reference
Cardiology imaging	3.60 (0.87, 14.94)
Electrophysiology	3.43 (1.19, 9.86)
Interventional cardiology	4.40 (1.63, 11.88)
Structural cardiology	5.99 (0.52, 68.99)
Heart failure	3.00 (0.40, 22.38)
AHP	2.86 (0.92, 8.91)
RN	2.54 (0.89, 7.21)
Experience, y	
0-5	Reference
>5-10	0.99 (0.47, 2.09)
>10	1.56 (0.84, 2.89)
Daily duration of lead wearing, h	
<2	Reference
>2-4	1.06 (0.51, 2.19)
>4-8	1.69 (0.86, 3.34)
>8	4.85 (1.25, 18.75)
Daily duration of lead wearing, h	
<4	Reference
>4	1.83 (1.12, 2.98)
Knowledge of radiation safety	
Very uncomfortable	Reference
Slightly uncomfortable	1.47 (0.59, 3.65)
Comfortable	3.35 (1.49, 7.56)
Very comfortable	1.79 (0.76, 4.23)
Knowledge to make ailments better	
Comfortable	Reference
Somewhat comfortable	1.23 (0.69, 2.15)
Somewhat uncomfortable	3.32 (1.42, 7.74)
Uncomfortable	4.35 (1.38, 13.79)
Percentage of fluoroless procedures	
<10%	Reference
>20%	1.06 (0.43, 2.59)
>50%	0.55 (0.22, 1.36)
Hours of exercise per week	
<2.5	Reference
2.5-4.5	0.92 (0.54, 1.57)
>4.5	1.57 (0.79, 3.12)

Values are OR (95% CI). Values of *P* < 0.05 were considered significant.

AHP = allied health professional; BMI = body mass index; RN = registered nurse.

**Table 4:** Risk Factors for Orthopedic Injuries

Age, y	
≤50	Reference
>50	2.19 (1.15, 4.16)
Male	1.49 (0.76, 2.92)
BMI, kg/m^2^	
<18.5	0.20 (0.05, 0.91)
18.5-24.9	Reference
25.0-29.9	0.64 (0.34, 1.22)
≥30	0.79 (0.36, 1.76)
Daily duration of lead wearing, h	
≤4 hours	Reference
>4 hours	2.39 (1.31, 4.39)
Hours of exercise per week	
<2.5	Reference
2.5-4.5	0.89 (0.47, 1.70)
>4.5	2.28 (0.96, 5.39)
Knowledge to make ailments better	
Comfortable	Reference
Somewhat comfortable	1.66 (0.85, 3.25)
Somewhat uncomfortable	4.30 (1.62, 11.45)
Uncomfortable	6.42 (1.67, 24.69)
Role	
Interventional	1.36 (0.66, 2.82)
EP	0.85 (0.39, 1.81)
Other^a^	Reference

Values are OR (95% CI). This table represents the multivariable regression model examining the association of several risk factors to orthopedic injuries in the total sample. Values of *P* < 0.05 were considered significant.

AHP = allied health professional; BMI = body mass index; RN = registered nurse; EP = electrophysiology; HF = heart failure.

a Other includes general, imaging, structural, HF, AHP, RN.

In the fully adjusted model for orthopedic injury related to work and requiring surgical intervention, individuals aged 71 to 80 had statistically significant increased odds of surgical orthopedic injury compared to those aged 41 to 50 (aOR = 9.40; 95% CI: 1.46-60.57; *P* = 0.02). In the same model, electrophysiologists had significantly lower odds of injury compared to ICs (aOR = 0.09; 95% CI: 0.02-0.52; *P* = 0.006). Similar findings were noted by comparing the general cardiology group (n = 21) with the remaining professionals (n = 285) (Table 5). These were considered controls as they had much lower daily lead-wearing time (≤4 hours: 87.5% vs 42.9%; *P* < 0.001), and radiation exposure with a lower rate of orthopedic injury (33.3% vs 63.5%; *P* = 0.009). Though they were significantly younger (*P* < 0.009), multivariable analysis across the entire sample revealed that age was not significantly associated with injury risk, while lead wearing >4 hours/day was significantly associated with orthopedic injury (*P* = 0.03), with a Hosmer-Lemeshow *P* value of 0.99 indicating good model fit and essentially no overfitting While both groups were not balanced in size, comparison between physicians and nonphysicians staff members did not result in statistically significant difference in orthopedic injury (aOR = 0.77, 0.37-1.6; *P* = 0.49) nor in orthopedic injury requiring surgery (aOR = 0.47, 0.10, 2.20; *P* = 0.34) (Table 6). Orthopedic injuries model had acceptable goodness-of-fit *P* values and area under the curves (Hosmer-Lemeshow *P* = 0.52 and 0.76, respectively).

**Table 5:** Comparisons of Various Characteristics and Outcomes by Role

	Total Cohort (N = 306)	General Cardiologist (n = 21)	Other Roles (n = 285)	*P* Value
Age, y				<0.001
20-30	21 (6.9)	3 (14.3)	18 (6.3)	
31-40	73 (23.9)	11 (52.4)	62 (21.8)	
41-50	76 (24.8)	1 (4.8)	75 (26.3)	
51-60	73 (23.9)	0 (--)	73 (25.6)	
61-70	42 (13.7)	1 (4.8)	41 (14.4)	
71-80	21 (6.9)	5 (23.8)	16 (5.6)	
Age, y				0.001
≤40	94 (30.7)	14 (66.7)	80 (28.1)	
>40	212 (69.3)	7 (33.3)	205 (71.9)	
Female	132 (43.1)	8 (38.1)	124 (43.5)	0.66
BMI, kg/m^2^				0.53
<18.5	8 (2.6)	1 (4.8)	7 (2.5)	
18.5-24.9	128 (41.8)	10 (47.6)	118 (41.4)	
25.0-29.9	113 (36.9)	8 (38.1)	105 (36.8)	
≥30	57 (18.6)	2 (9.5)	55 (19.3)	
Years of experience				<0.001
0-5	53 (17.3)	12 (57.1)	41 (14.4)	
>5-10	57 (18.6)	3 (14.3)	54 (18.9)	
>10	196 (64.1)	6 (28.6)	190 (66.7)	
Daily duration of lead wearing (hours)	(n = 282)	(n = 16)	(n = 266)	<0.001
≤4 hours	128 (45.4)	14 (87.5)	114 (42.9)	
>4 hours	154 (54.6)	2 (12.5)	152 (57.1)	
Prior estimated daily duration lead (hours)	(n = 113)	(n = 13)	(n = 100)	0.23
<2	35 (30.9)	7 (53.9)	28 (28.0)	
>2-4	28 (24.8)	3 (23.1)	25 (25.0)	
>4-8	35 (30.9)	3 (23.1)	32 (32.0)	
>8	15 (13.3)	0 (--)	15 (15.0)	
Cataracts/cancer	(n = 299)	(n = 21)	(n = 278)	0.76
Yes	50 (16.7)	4 (19.1)	46 (16.6)	
Radiation safety knowledge	(n = 296)	(n = 20)	(n = 276)	0.002
Very uncomfortable	30 (10.1)	0 (--)	30 (10.9)	
Slightly uncomfortable	51 (17.2)	9 (45.0)	42 (15.2)	
Comfortable	139 (46.9)	10 (50.0)	129 (46.7)	
Very comfortable	76 (25.7)	1 (5.0)	75 (27.2)	
Knowledge to make ailment better	(n = 275)	(n = 18)	(n = 257)	0.07
Comfortable	86 (31.3)	4 (22.2)	82 (31.9)	
Somewhat comfortable	119 (43.3)	7 (38.9)	112 (43.6)	
Somewhat uncomfortable	45 (16.4)	7 (38.9)	38 (14.8)	
Uncomfortable	25 (9.1)	0 (--)	25 (9.7)	
Prior orthopedic injury				
Yes	188 (61.4)	7 (33.3)	181 (63.5)	0.009
Required surgery for orthopedic injury	(n = 181)	(n = 5)	(n = 176)	--
Yes	22 (12.2)	0 (--)	22 (12.5)	
Pain level for orthopedic injury	(n = 173)	(n = 5)	(n = 168)	0.002
<3	23 (13.3)	4 (80.0)	19 (11.3)	
3-7	108 (62.4)	1 (20.0)	107 (63.7)	
>7	42 (24.3)	0 (--)	42 (25.0)	
Resolution of orthopedic injury	(n = 177)	(n = 4)	(n = 173)	1.00
Yes	90 (50.9)	2 (50.0)	88 (50.9)	
Prior cancer history	(n = 287)	(n = 18)	(n = 269)	0.02
Yes	10 (3.5)	3 (16.7)	7 (2.6)	
Percentage of fluoroless procedures	(n = 241)	(n = 10)	(n = 231)	0.41
<10%	195 (80.9)	7 (70.0)	188 (81.4)	
>20%	46 (19.1)	3 (30.0)	43 (18.6)	
Hours of exercise per week	(n = 283)	(n = 16)	(n = 267)	0.79
<2.5	116 (40.9)	8 (50.0)	108 (40.5)	
2.5-4.5	110 (38.9)	5 (31.3)	105 (39.3)	
>4.5	57 (20.1)	3 (18.8)	54 (20.2)	
History of pregnancy	(n = 118)	(n = 6)	(n = 112)	0.34
Yes	86 (72.9)	3 (50.0)	83 (74.1)	
Back/neck pain during pregnancy	(n = 84)	(n = 3)	(n = 81)	1.00
Yes	47 (55.9)	2 (66.7)	45 (55.6)	
Pregnancy complications exacerbated by work	(n = 78)	(n = 2)	(n = 76)	0.29
Yes	12 (15.4)	1 (50.0)	11 (14.5)	
Opportunity to adjust procedure load	(n = 70)	(n = 2)	(n = 68)	1.00
Yes	33 (47.1)	1 (50.0)	32 (47.1)	
Lead wearing during pregnancy	(n = 74)	(n = 3)	(n = 71)	1.00
Yes	51 (68.9)	2 (66.7)	49 (69.0)	
Opportunity to decrease work hours	(n = 73)	(n = 3)	(n = 70)	0.53
Yes	16 (21.9)	1 (33.3)	15 (21.4)	
Fluoroless procedures performed	(n = 69)	(n = 3)	(n = 66)	1.00
Yes	23 (33.3)	1 (33.3)	22 (33.3)	

Values are n (%), unless otherwise indicated. This table compares the characteristics of general cardiologist vs other roles. Values of *P* < 0.05 were considered significant.

BMI = body mass index.

Pregnancy complications exacerbated by working include increased musculoskeletal pain, syncope, uterine bleeding, and miscarriage.

**Table 6:** Comparison Between Physicians and Nonphysicians

	Total Cohort (N = 306)	Physicians (n = 213)	Nonphysicians (n = 93)
Age, y			
≤50	170 (55.6)	111 (52.1)	59 (63.4)
>50	136 (44.4)	102 (47.9)	34 (36.6)
Female	132 (43.1)	63 (29.6)	69 (74.2)
BMI, kg/m^2^			
<18.5	8 (2.6)	5 (2.4)	3 (3.2)
18.5-24.9	128 (41.8)	94 (44.1)	34 (36.6)
25.0-29.9	113 (36.9)	80 (37.6)	33 (35.5)
≥30	57 (18.6)	34 (15.9)	23 (24.7)
Years of experience			
0-5	53 (17.3)	33 (15.5)	20 (21.5)
>5-10	57 (18.6)	38 (17.8)	19 (20.4)
>10	196 (64.1)	142 (66.7)	54 (58.1)
Daily duration of lead wearing, h	(n = 282)	(n = 200)	(n = 82)
≤4 hours	128 (45.4)	103 (51.5)	25 (30.5)
>4 hours	154 (54.6)	97 (48.5)	57 (69.5)
Previous estimated daily duration of lead wearing, h	(n = 113)	(n = 80)	(n = 33)
<2	35 (30.9)	26 (32.5)	9 (27.3)
>2-4	28 (24.8)	21 (26.3)	7 (21.2)
>4-8	35 (30.9)	25 (31.3)	10 (30.3)
>8	15 (13.3)	8 (10.0)	7 (21.2)
Cataracts/cancer	(n = 299)	(n = 209)	(n = 90)
Yes	50 (16.7)	43 (20.6)	7 (7.8)
Radiation safety knowledge	(n = 296)	(n = 206)	(n = 90)
Very uncomfortable	30 (10.1)	19 (9.2)	11 (12.2)
Slightly uncomfortable	51 (17.2)	37 (17.9)	14 (15.6)
Comfortable	139 (46.9)	98 (47.6)	41 (45.6)
Very comfortable	76 (25.7)	52 (25.2)	24 (26.7)
Knowledge to make ailment better	(n = 275)	(n = 194)	(n = 81)
Comfortable	86 (31.3)	56 (28.9)	30 (37.0)
Somewhat comfortable	119 (43.3)	86 (44.3)	33 (40.7)
Somewhat uncomfortable	45 (16.4)	34 (17.5)	11 (13.6)
Uncomfortable	25 (9.1)	18 (9.3)	7 (8.6)
Percentage of fluoroless procedures	(n = 241)	(n = 169)	(n = 72)
<10%	195 (80.9)	139 (82.3)	56 (77.8)
>20%	46 (19.1)	30 (17.8)	16 (22.2)
Hours of exercise per week	(n = 283)	(n = 199)	(n = 84)
<2.5	116 (40.9)	77 (38.7)	39 (46.4)
2.5-4.5	110 (38.9)	83 (41.7)	27 (32.1)
>4.5	57 (20.1)	39 (19.6)	18 (21.4)
Select outcomes			
Orthopedic injury			
Yes	188 (61.4)	135 (63.4)	53 (56.9)
Surgery for orthopedic injury	(n = 181)	(n = 129)	(n = 52)
Yes	22 (12.2)	15 (11.6)	7 (13.5)
Pain level for orthopedic injury	(n = 173)	(n = 125)	(n = 48)
<3	23 (13.3)	16 (12.8)	7 (14.6)
3-7	108 (62.4)	72 (57.6)	36 (75.0)
>7	42 (24.3)	37 (29.6)	2 (10.4)
Resolution of orthopedic injury	(n = 177)	(n = 127)	(n = 50)
Yes	90 (50.9)	68 (53.5)	22 (44.0)
Cancer perceived as work-related	(n = 287) 10 (3.5)	(n = 201) 8 (3.9)	(n = 86) 2 (2.3)

Values are n (%), unless otherwise indicated. This table uses chi-square or Fisher tests to compare risk factors and outcomes between physicians and nonphysician subgroups.

BMI = body mass index.

### Cataract and malignancy

Cataracts were reported in 16% of the respondents and brain, bone, breast, or lung cancer reported in 3.5%. EP/CCL workers over the age 41 to 50 and those uncomfortable with radiation-safety knowledge showed significant association with cataract or cancer (aOR = 10.62; 95% CI: 1.32-85.58; *P* = 0.03, aOR = 4.19; 95% CI: 1.38-12.74; *P* = 0.01) in the multivariable analysis (Tables 7 and 8). Although the OR for cataract or cancer in the interventional group was 3.29 (95% CI: 1.16-9.33; *P* = 0.03) before Bonferroni correction, the association did not remain statistically significant after adjustment (OR: 3.29; 95% CI: 0.92-11.75; *P* = 0.08). Compared to nonphysicians, physicians had nearly 4 times the odds of developing cataracts or malignancies (aOR: 3.88; 95% CI: 1.29-11.57; *P* = 0.02, Hosmer-Lemeshow *P* = 0.89), when adjusted for age, sex, BMI, years of experience, daily-duration lead wearing, radiation-safety knowledge, and knowledge to make ailment better (Table 6).

**Table 7:** Risk Factors for Cataracts and/or Cancer Development in EP/CCL Staff

Age, y	
≤50	Reference
>50	7.56 (3.14, 18.23)
Male	1.53 (0.59, 3.96)
BMI, kg/m^2^	
<18.5	1.73 (0.19, 16.13)
18.5-24.9	Reference
25.0-29.9	1.44 (0.63, 3.30)
≥30	2.11 (0.75, 5.87)
Daily duration lead wearing, h	
≤4 hours	Reference
>4 hours	1.07 (0.49, 2.29)
Hours exercise per week	
<2.5	Reference
2.5-4.5	0.71 (0.29, 1.68)
>4.5	2.43 (0.89, 6.65)
Knowledge to make ailment better	
Comfortable	Reference
Somewhat comfortable	1.80 (0.69, 4.74)
Somewhat uncomfortable	1.43 (0.41, 5.02)
Uncomfortable	3.88 (0.98, 15.34)
Role	
Interventional	3.29 (1.16, 9.33)
EP	2.17 (0.66, 7.08)
Other^a^	Reference

Values are OR (95% CI). This table lays out the ORs for risk factors for cataracts/cancer in the survey cohort as determined by univariable regression model.

AHP = allied health professional; BMI = body mass index; EP = electrophysiology; HF = heart failure; RN = registered nurse.

a Other includes general, imaging, structural, HF, AHP, RN.

**Table 8:** Risk Factors for Cataracts and Cancer in Multivariable Regression Models

Age, y	
41-50	Reference
51-60	1.68 (0.53, 5.27)
61-70	6.22 (1.74, 22.21)
71-80	17.11 (3.13, 93.52)
Female	1.24 (0.42, 3.65)
BMI, kg/m^2^	
<18.5	0.66 (0.08, 5.09)
18.5-24.9	Reference
25.0-29.9	1.85 (0.71, 4.83)
≥30	2.17 (0.69, 6.80)
Years of experience	
>5-10	1.94 (0.47, 8.03)
>10	Reference
Daily lead wearing, h	
≤4 hours	Reference
>4 hours	1.52 (0.60, 3.86)
Radiation safety knowledge	
Very uncomfortable	4.19 (1.38, 12.74)
Slightly uncomfortable	1.65 (0.43, 6.27)
Comfortable	Reference
Very comfortable	1.03 (0.34, 3.14)
Exercise (hours/week)	
<2.5	Reference
2.5-4.5	0.48 (0.18, 1.31)
>4.5	2.22 (0.72, 6.82)

Values are OR (95% CI). This table lays out the ORs for risk factors for cataracts/cancer in the survey cohort as determined by multivariable regression model.

BMI = body mass index.

### Pregnancy-specific injuries

Among respondents, 32.1% reported having been pregnant during their career. Of those, 55.8% experienced neck or back pain during pregnancy, and 41.5% reported that their occupational duties worsened this discomfort. Additionally, 15% self-reported that the pregnancy complications, such as uterine bleeding, syncope, and miscarriage, were exacerbated by work-related factors. Workplace accommodations were limited: 52.1% had no opportunity to adjust procedural load, 76% were unable to reduce work hours, and 68.4% wore lead during pregnancy. During pregnancy, only 33% performed fluoroless procedures. These findings suggest a substantial physical burden during pregnancy, with limited flexibility and adjustments in occupational demands. Comparison between pregnant and nonpregnant women was limited by small sample size but nonetheless there was a trend towards higher orthopedic injuries.

### Educational video impact

A second survey was distributed to assess changes in knowledge, awareness, and implementation of safe practices. There were 99 responses to the second survey (2% response rate). Of these respondents, 34.1% recalled viewing the 20-minute educational video 3 months prior. Of these, the majority reported feeling either very comfortable (48.3%) or comfortable (41.4%) with their knowledge of radiation safety. Additionally, 75.9% indicated successful implementation of the ergonomic techniques demonstrated in the video into their daily practice.

## Discussion

This study provides updated insights into occupational hazards among EP/CCL professionals. It aimed to identify risk factors for MSK injuries and radiation-related medical conditions, and to assess the effect of an educational intervention focused on radiation protection and knowledge of ergonomic strategies. It showed: 1) MSK pain or injury perceived to be work related remains highly prevalent, particularly among those with daily duration of lead use> 4 hours; 2) MSK pain and other medical complications such as syncope, uterine bleeding, and miscarriage occurred in 15% and 10% of pregnant women, respectively, with limited accommodations; and 3) a brief educational video was perceived as helpful in addressing physical strain and mitigating risks for EP/CCL professionals. The results are unique in that they showed that wearing lead for >4 hours per day was significantly related to a higher rate of orthopedic injuries, even after correction for possible confounding factors. This novel finding allows for potentially avoiding injury by limiting prolonged lead wearing to <4 hours/day, if possible. The second important finding is that despite the advancements in technology over the last decade, orthopedic injury rates remain high and comparable to those reported 10 years ago, continuing to impact healthcare professionals missed worked days and career changes.^2^^,^^4^^,^^5^^,^^8^

With regards to the occurrence of cataracts and cancer, this study highlights age and perceived low radiation safety knowledge as risk factors. Advancing age was the strongest factor associated with cataract and cancer, with individuals aged 71 to 80 having 17 times the risk compared to those aged 41 to 50. These findings are consistent with prior literature linking cumulative radiation exposure and age-related susceptibility to ocular and oncologic outcomes.^10^ In this cohort, neither cancer nor cataracts were identified as occupationally linked outcomes most likely due to the cross-sectional nature of the study and lack of longitudinal follow-up; however, age emerged as associated with both outcomes. These findings underscore the need for targeted radiation safety education and potential procedural modifications, particularly among high-risk roles and older professionals. Physicians may experience a higher cumulative exposure to ionizing radiation over time due to their closer and more frequent proximity to the radiation source compared to other health care workers in the procedural room. This increased exposure may partly explain the observed higher prevalence of cataracts and certain malignancies in physicians vs nonphysicians.^11^

Respondents reported increased MSK pain during pregnancy. During pregnancy, hormonal ligament laxity and the anterior shift in the center of gravity accentuate lumbar lordosis predisposing individuals to back pain.^12^ A 15% complication rate during pregnancy, including uterine bleeding and miscarriage, was reported by EP/CCL respondents, aligning with findings from studies in the general population.^13^ This rate can be even higher among female surgeons—in one survey, 31% of respondents reported work-related pregnancy complications.^14^ For pregnant females, more frequent breaks during prolonged cases can mitigate some of these adverse events. Fluoroless procedures could potentially be of use as well in this population, helping to decrease radiation exposure to the fetus. Fluoroless procedures have gained popularity in EP, and the increased use of electroanatomic mapping systems and intracardiac echocardiography has significantly reduced fluoroscopy time.^15^ Finally, nonphysician CVT members, comprising 30% of the responses, showed they have an equally high risk of occupational injuries. This study showed that technologists and nurses have an equally high risk of occupational MSK, cataract, or cancer risk as physicians, which has been reported previously.^8^ These findings reinforce the importance of broad-based preventive strategies that include all team members working in the EP/CCL environment.

### Study Limitations

Our study is based on survey data, and like other survey-based studies, there is a risk of selection bias and reliance on self-reporting of various medical conditions, without objective documentation. In addition, the survey responses may reflect location-based clustering, with staff from certain sites participating at higher rates than others. However, this could not be specifically determined because the survey was anonymous. There is also a lack of data obtained for specific variables, such as a direct assessment of radiation dose, the number of days off work because of injuries as well as other lifestyle factors that might be affecting orthopedic injuries, cataracts, and malignancies. Furthermore, because of the cross-sectional design of the survey and the relatively short follow-up period, conclusions regarding causality of chronic low-dose ionizing radiation to stochastic risks like cancer and cataract are limited. Those CCL workers who did develop cancer may have transitioned into a new job or retired altogether and would not be captured by this survey. This would also hold true for workers who developed work-limiting MSK injuries who thus may have chosen to stop working in the CCL altogether. This would bias the results in favor of the null hypothesis.

The overall response rate was estimated and could not be accurately assessed, and several responses had to be omitted because of incomplete data. The controls used were general cardiologists who answered the survey and have limited interventional exposure. This group might not mimic the general population and no propensity score matching could be performed due to the small group size of each covariate. Finally, the second survey respondents' rate was low so the efficacy of an educational video cannot be generalized.

## Conclusions

In summary, occupational hazards in the EP/CCL underscore the importance of comprehensive radiation protection measures and ergonomic interventions. Despite decades of advancements in the EP/CCL, the prevalence of occupational risk on EP/CCL professionals remains the same. Continued research and implementation of evidence-based strategies are essential to safeguard the health and well-being of these healthcare professionals. This paper might serve as a pillar for call to action to reduce the occupational hazards in EP/CCL.

Perspectives **COMPETENCE IN PATIENT CARE AND PROCEDURAL SKILLS:** Occupational health hazards remain prevalent in cardiac catheterization and electrophysiology laboratories despite advances in technology. Knowledge of best practices may help to mitigate injury and harm. This can be done in the format of a short educational video, such as the one used in this study focusing on basic radiation safety principles, optimal ergonomics in the EP/CCL and best practices during pregnancy.

**TRANSLATIONAL OUTLOOK:** Validating the feasibility and efficacy of simple 30-second stretching exercises can be envisioned and performed during cases to help prevent injury without compromising patient care.

## Funding support and author disclosures

This study is funded by the ACC chapter/section grant. The authors have reported that they have no relationships relevant to the contents of this paper to disclose.
